# Vaccination decisions and social capital in Japan

**DOI:** 10.1016/j.ssmph.2025.101769

**Published:** 2025-02-26

**Authors:** Toshihiro Okubo, Ilan Noy

**Affiliations:** a Keio University, Japan; b Victoria University of Wellington, New Zealand; c Gran Sasso Science Institute, Italy

**Keywords:** Vaccine, COVID-19, Social capital, Trust

## Abstract

The COVID-19 vaccines played a pivotal role in safeguarding many people. Yet, vaccine hesitancy remained a significant barrier to increasing coverage rates, as many high-income countries faced prolonged vaccine refusal campaigns. In Japan, vaccine doses were administered under a reservation system accessible via a website and by phone. Achieving a high vaccination coverage for a vaccine that was offered at no financial cost was surprisingly difficult in Japan as well. In many countries, vaccine hesitancy during the pandemic has been closely related to people's trust in their governments given governments' controversial social distancing mandates. In Japan, lockdowns were voluntary, and vaccinations were also not mandated. As there were no significant political conflicts about the government's policies, vaccination acceptance was influenced by more basic tenets, and we focus here on social capital, defined as cohesive links that enable a society to function effectively. Social capital, in this context, refers to community trust, collaboration, and engagement that create social bonds between individuals and society. Using a uniquely large survey, administered repeatedly through the years of the pandemic, we mostly find support, for the hypothesis that social capital matters for the vaccination decision; and that it matters even once we control for institutional trust (especially trust in the medical system). However, this general association between trust in other community members, belief in the willingness of community members to engage in reciprocal assistance, and belief in the more general willingness of the community to support individuals, were all associated differently with the vaccination decision, and with the views expressed about the vaccinations. From a policy perspective, this suggests that intra-community trust (i.e., bonding social capital), is important even in contexts when trust in governmental is not a significant concern.

## Highlights

• The COVID-19 vaccines played a pivotal role in safeguarding populations. Yet, vaccine hesitancy remained a significant barrier to increasing coverage rates. In Japan, achieving a high vaccination coverage for a vaccine that was offered at no financial cost was still difficult.
• Using a unique, large, survey, administered repeatedly through the years of the pandemic, we found support the hypothesis that social capital matters for the vaccination decision. Social capital refers to community trust, collaboration, and engagement that create social bonds between individuals and society.
• The association between trust in other community members, trust in the willingness of community members to engage in reciprocal assistance, and trust in the more general willingness of the community to support individuals, were all associated differently with the vaccination decision.

## 1 Introduction

During the global COVID-19 pandemic, vaccines have emerged as the primary, cost-effective public health tool to prevent serious health and life endangering consequences in societies across the world. The COVID-19 vaccines thus played a pivotal role in safeguarding populations from the virus and its affects. Their swift development and deployment in late 2020 clearly helped to contain the spread and severity of the COVID-19 pandemic. Retrospective statistical analysis found the vaccine prevented an estimated 1.6 million additional deaths in Europe alone (Meslé et al., 2024) or 2.3 million globally (Agrawal, & Whaley, 2023) but computer modelling of the disease spread typically identified much bigger numbers (up to 20 million deaths in the vaccine's first year; in Watson et al., 2022). Of course, vaccines are deployed not only to fight against global pandemics, and their role in fighting epidemics or more generally infectious diseases has long been central for public health.

Yet, despite a long experience with many safe vaccines, and research establishing the safety and efficacy of the COVID-19 vaccinations, vaccine hesitancy remained a significant barrier to increasing coverage rates to levels that can induce herd immunity (Dubé and MacDonald, 2022a, Dubé and MacDonald, 2022b). Many high-income countries faced prolonged vaccine refusal campaigns for a variety of vaccines and infectious diseases, with acceptance and uptake rates widely differing across countries (Wang et al., 2022). For COVID-19, many countries have not achieved the recommended coverage rate for the initial vaccination protocol (Mathieu, Ritchie, Ortiz-Ospina et al., 2021) – a coverage rate that significantly affects the aggregate spread of the disease and is thus important for population health. Previous studies have even found that people prefer vaccines made in-country (Kawata & Nakabayashi, 2021; Kreps et al., 2020; Motta, 2021; Schwarzinger et al., 2021; Sun et al., 2020). This home-bias may actually imply that people trust their own government and medical system, possibly more than they trust, for example, international authorities like the World Health Organization.

As a reminder, in March 2020 the World Health Organization declared a pandemic in response to the spread of a highly infectious novel coronavirus that first emerged in December 2019. Vaccination development began very quickly, and the first vaccine was already authorised for emergency use by the end of 2020. In Japan, the country which we focus on, the vaccine was approved in February 2021 for the Pfizer vaccine and in May 2021 for the Moderna, AstraZeneca, and Johnson & Johnson vaccines. Early doses started to be used on February 17th, 2021, for medical workers (approximately 4.7 million people), then for people 65 years or older from April 12th, 2021 (approximately 36 million people), and for all people 18 years or older from June 1st, 2021.^1^

Vaccine doses were administered by municipality offices, healthcare centers, and some hospitals under a reservation system accessible via a website and by phone. In addition, the Japanese government allowed some universities and large companies to provide vaccines. As public concern and fear increased, vaccination centers were overwhelmed with demand and thus many people had to wait a few weeks to a few months for their vaccination. This waiting period, however, happened long before the survey we rely on the gauge vaccine behavior.

Achieving a high vaccination coverage for vaccines that are offered at no financial cost was still surprisingly difficult in Japan, as was the case in many other countries. Understanding what determined people's acceptance of the vaccine remains essential in addressing vaccine hesitancy for COVID-19, and any other vaccine-preventable diseases. In many countries, vaccine hesitancy during the pandemic has been closely related to people's trust in their governments. Governments the world over have played an outsized and novel role in responding to this pandemic, with stringent restrictions placed in many places on personal movement, work practices, and socializing. This aggressive and unprecedented response led, in many places, to a ‘trust crisis’ between many and their government.

In Japan, however, there were few strict regulations or penalties for individual behavior, as the lockdowns were voluntary. The Japanese government requested the public's cooperation rather than demanded or mandated it. That may be one reason why the general COVID-19 response policy did not generate, in Japan, a violent backlash as it did in many other places. Vaccinations were also neither mandatory nor heavily enforced; for example, through restrictions about what activities the unvaccinated can be involved in (Cameron-Blake et al., 2023). In spite of it, the vaccination rates achieved within a comparably short period were high. Elderly people started the first dose in April 2021 and around 80% of them finished the second dose by the end of July 2021. For all other people above age 18, whose vaccination started in June 2021, around 77% of them finished the second dose as of the end of November 2021. Regarding anticipatory vaccine hesitancy, around 50% of the Japanese people were eager to take vaccination, around 20–30% were indecisive and 10–20% hesitated about it as of February 2021, before the first doses of the vaccine started (Kadoya et al., 2021; Nomura et al., 2021; Okamoto et al., 2022; Okubo et al., 2021; Okubo et al., 2021, Okubo et al., 2021).^2^

We hypothesize that, in Japan, where there were no significant political conflicts about the voluntary lockdowns, and vaccination in particular, vaccination behavior was influenced by more basic tenets. In particular, we focus here on social capital, defined as cohesive resources that enable a society to function effectively. These social capital ties are accessed by individuals as a result of membership of certain groups and communities. Aldrich (2012) differentiates between bonding, bridging, and linking social capital in the context of disasters. Here we focus on the various aspects of the social ties that Aldrich (2012) defines as bonding social capital.

In order to identify the association between social capital and the willingness to vaccinate, we also need to control for other aspects of each respondent that may affect their acceptance of the vaccine. In particular, we also control for the health condition of the individuals considering a vaccination, other non-cognitive factors associated with them, and their socio-demographic traits.

Social capital, in this context, refers to community trust, collaboration, and engagement that create social bonds between individuals and society (e.g. Bourdieu, 1986; Coleman, 1988; Putnam et al., 1993). Social capital can facilitate and influence decision-making about vaccination through the exchange of information, by identifying problems and concerns, and by ameliorating the emergence of conflicting or false information in communities. Evidence that supports this conjecture of a link between social capital and health-related choices were identified for H1N1 vaccination decisions by parents for their children (Jung et al., 2013), for protective behaviors against influenza in Taiwan (Chuang et al., 2015), and, using aggregate community-level vaccination rate data, for the average vaccination uptake in the Lombardy region of Italy during the COVID-19 pandemic (Buonanno et al., 2023). In Japan, similar association was identified in both aggregate and micro-level data for measles vaccinations (Nagaoka et al., 2012; Nawa & Fujiwara, 2019) and pneumococcal vaccination in the large sample of the Japan Gerontological Evaluation Study (Iwai-Saito et al., 2021).

Several studies assessed the relationship between social capital and COVID-19 vaccination decisions using survey data in various countries. Several focused on either bonding social capital (i.e., relationships between family, friends or in a community), and bridging social capital (i.e., connections formed between different communities). In this literature, the decision to get vaccinated was found to be positively associated with social trust (Kreps & Kriner, 2023; Oshio & Ping, 2023), social cohesion (Kim & Lim, 2024; Machida et al., 2022; Oshio & Ping, 2023), and perceived social support (Datta et al., 2023). Vaccination decisions also appear to be influenced by opinions about the vaccine and by peer-pressure (i.e., the influence of family, friends, and neighbors) (Bernados & Ocampo, 2022; Latkin et al., 2022), social norm (Schmidt-Petri et al., 2022), and monetary incentives (Yamamura et al., 2025).^3^

Other studies focus on the effects of linking social capital (i.e., the vertical connections to the political center). These measured trust in institutions such as government, healthcare or media institutions. Here, the decision to get vaccinated was found to be positively associated with trust in the government (Bajos et al., 2022; Cvjetkovic et al., 2022; Latkin et al., 2023; Lomeli et al., 2023; Oshio & Ping, 2023; Rivera, 2023; Viskupič et al., 2022), trust in healthcare providers, medical experts, scientists, and vaccine approval systems (Bajos et al., 2022; Cao et al., 2024; Cvjetkovic et al., 2022; Lomeli et al., 2023; Viskupič et al., 2022), and trust in official information sources and the traditional media (Cao et al., 2024; Juarez, Kang, et al., 2022, Juarez et al., 2022, Juarez et al., 2022; Latkin et al., 2023).

Regarding COVID-19 in Japan, public debates about vaccination mainly focused on safety concerns, scheduling and prioritization issues, and vaccine supply (imports and distributional logistics), and not on contentious mandates or lockdowns. This stands in stark contrast to other countries where the vaccination campaign was often seen as a proxy referendum about the reigning government's policy choices during the pandemic's initial phase. In many cases, it took on importance as an ideological litmus test of fealty to the current leadership in office.^4^

It is important to note that the unique Japanese background for these dynamics is deeply rooted in the Japanese Constitution.^5^ After Japan's defeat in World War II, the country was occupied by the U.S. military, which sought to eliminate the threat of any future Japanese imperial aspiration. The constitution enacted in 1947 explicitly states that the government, including the police, cannot force people to act in ways that infringe on people's human and property rights.^6^ Thus, for constitutional reasons, Japan was unable and probably unwilling to impose mandatory lockdowns that included penalties or sanctions, as was done in almost all other high-income countries, and in many middle- and low-income ones, as well.

The Japan case thus provides an opportunity not to focus on trust in government and other institutions of power (linking social capital) as determinants of vaccine hesitancy.^7^ Rather, we can focus here on people's perceived social connections (bonding social capital), and specifically their views about their commitments to their community and the community's commitment to them. This paper thus seeks to explore how effective was Japan's vaccination campaign, which relied on the power of these social connections, while it avoided strict lockdowns. This is the main contribution of this analysis of the Japanese case, compared to jurisdictions where these two aspects - trust in government and other institutions, and the strength of social connections were muddled.

In the following pages, we first describe the unique dataset we use, a repeat large-scale panel survey conducted in Japan over multiple years (section 2), the hypotheses we test (section 3), and the methodology we use to empirically examine the association between vaccination decisions and perceived social capital (section 4). We then describe and discuss our results (section 5) and conclude with some thoughts about future directions and the policy implications of our findings.

## 2 Data and variables to use

### 2.1 Panel survey, data, and variables to use

**Fig. 1:**
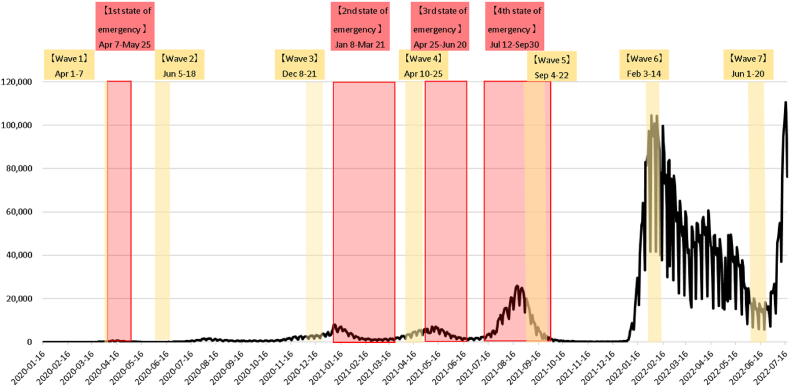
COVID-19 infections, emergency state, and survey waves (until wave 7, July 2022) (Okubo, 2022).

The data used in this study comes from the Okubo-NIRA survey (Okubo and NIRA, 2020a,b,c, 2021a,b, 2022), “Questionnaire Survey on the Effects of the Spread of COVID-19 on Telework-based Work Styles, Lifestyle, and Awareness.” This is a panel survey conducted in Japan over eleven waves of approximately 10,000 workers in a randomly stratified sample as of November 2024.^8^ The survey asked about worker's characteristics, their lives, and their working environments. The first wave was conducted in March 2020, just before the spread of the epidemic to Japan and the declaration of the first state of emergency in the country in April 2020. As of writing, the latest wave was conducted in July 2024. See Fig. 1 for details about the timing of survey waves and the aggregate number of COVID-19 infections in Japan. Many respondents repeatedly responded to the survey over multiple waves.^9^

For the purposes of this study, we primarily use data from wave 6 (February 2022), which focused on vaccination. This wave collected information on the number of vaccine doses received, the vaccine's side effects, opinions and perceptions about the vaccination programme, the respondents' COVID-19 infection status, their pre-existing illnesses and mental health during the pandemic, and their family's health. Wave 6 took place around the time when the third vaccine dose was introduced in Japan. Although wave 5 (September 2021) also collected some vaccination-related data, it only asked about the number of doses and the willingness to be vaccinated, and these questions were also asked in wave 6. Therefore, wave 6 serves as the main data source for our study, but wave 5 is used to investigate the change of vaccination behaviors between the two waves, as a complementary analysis. Out of total sample of wave 6 (10,113), 8058 respondents also participated in wave 5.

Additionally, we incorporate data from wave 4 (April 2021), which examined the willingness to commute to work. The need or desire to commute in public transportation may also be positively related to individuals' willingness to receive vaccinations. Out of the total sample of wave 6 (10,113), 6945 respondents participated also in wave 4.

In our setup, the declared vaccination choice is the dependent variable – our focus is explaining this choice. The dependent variable is regressed on two sets of explanatory variables: (1) basic socio-economic traits and (2) non-cognitive and health conditions. The basic socio-economic traits we can include are age, gender, income, occupation, employing firm size, employment status, educational background, ICT skills, the proportion of new COVID-19 cases in the respondent's residential municipality, teleworking, and other changes in life circumstances (time allocation, income, and life satisfaction).^10^ The non-cognitive and health factors consist of variables related to social capital (our focus), the respondent's health condition (including COVID-19 infection and pre-existing illnesses), fear of infection, previous side effects from vaccination (if applicable), family infection status, and changes in trust toward central/local government and the medical system from the pre-pandemic to the pandemic period.

The survey first asked about the respondents’ characteristics; this included gender, age (scaled by decade), annual income in 2021 (scaled by 500 thousand yen), education (final degree ranged from junior high school = 1, high school = 2, college = 3, university = 4, master = 5, to Ph.D. = 6), and ICT skills required for work (0 = no need, 1 = preliminary, 2 = middle, 3 = advanced), occupation (38 categories), employment status (6 categories, e.g. regular, non-regular), and firm size (6 categories) (See Appendix Table A1 for more details).

### 2.2 Vaccination behavior

The survey asked respondents about the number of vaccine doses they had received and their willingness to be vaccinated, offering six options. This forms the dependent variable in our estimations (“Vaccination”). After wave 5 (September 2021), during the period of wave 6 (February 2022), the third dose of the vaccine began to be administered. Respondents were asked to choose one of the following six options regarding their willingness to be vaccinated and their vaccination behavior: You have received the third dose (=5); you want to receive the third dose but have not yet (=4); you have received the second dose but do not intend to receive the third (=3); you have received the first dose but do not intend to receive the second (=2); you want to receive the first dose but have not yet (=1); you do not intend to receive any vaccination (=0). A higher value indicates a greater willingness to be vaccinated.

Table 1 describes the responses of all those who answered both wave 5 and wave 6 of the survey. We point out there is a small number of respondents (114 out of 7562) whose responses between the two surveys were inconsistent. Otherwise, we observe that the majority of people have already taken the second dose by wave 5, and of these, about 90% intended to take the third dose as well (or have already done so). About 10% of the respondents have indicated they do not intend to vaccinate at all.

**Table 1:**
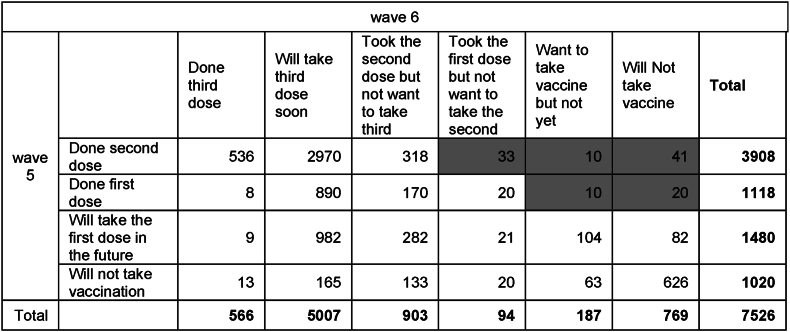
Vaccine Behavior.

Note: The shaded results are not internally consistent in their responses.

### 2.3 Opinions about vaccination

The survey asked respondents about their opinion about the COVID-19 vaccination. For each statement, respondents were asked to choose whether they "agree," "disagree," or are "neutral." We code our perception variables as (agree = 1, neutral = 0, disagree = −1) for these 10 items.

1. Vaccination reduces your own risk of infection (self-protection)
2. Vaccination can protect your family (people around you) from infection (protect others)
3. The safety of the vaccine has not been confirmed (safety concern)
4. Worried about side effect from vaccination (side effects concern)
5. No time to go for vaccination (time concern)
6. Difficult to make and keep appointments for vaccinations (reservation system)
7. Do not infect yourself or do not become seriously ill (no infection)
8. COVID is not serious enough to warrant vaccination (no covid)
9. basic infection prevention measures are sufficient without vaccination (mask)
10. Many people have already been vaccinated and so no need to be vaccinated (no need)

### 2.4 Bonding social capital

There were many previous studies on social capital since Hanifan (1916) coined the term – e.g., Putnam et al. (1993), Coleman (1988), and Bourdieu (1986). Social capital refers to resources of community trust, collaboration, and regional engagement that create social bonds between individuals and society, which functions to enhance efficiency within society.^11^ In our context, we hypothesize that bonding social capital resulted in a rapid and high take-up of vaccination, relying on mutual cooperation and without resorting to mandates and the application of power by the state.

We construct three variables to measure bonding social capital. Following the Putnam et al. (1993) framing, we define it as: (1) trust in other people (“Trust people”); (2) willingness and belief in mutual help (“Reciprocity”); and (3) disposition to cooperate and contribute to one's community (“Cooperation”). In the wave 6 questionnaire, trust in other people is measured by the average score of two statements: “In general, we can trust other people,” and “We can trust our neighbors.” Mutual help is measured by the average score of two statements: “People must help each other,” and “If you help others, they will help you when needed.” Cooperation is measured by the response to the statement: “Local resources must be protected through collaboration of all local residents.”

**Fig. 2:**
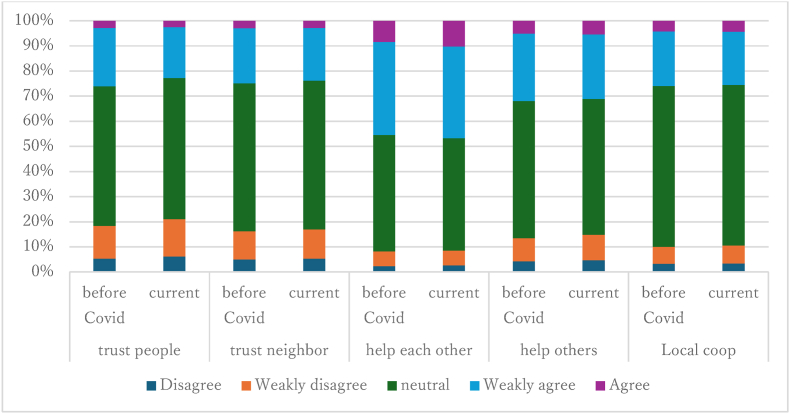
Responses about bonding social capital in wave 6.

For each statement, respondents were asked to state if they strongly agree (=5), agree (=4), neutral (=3), disagree (=2), or strongly disagree (=1). We then take average score of these three dimensions of social capital - i.e., trust, mutual help, and cooperation.^12^ Fig. 2 describes the breakdown of the survey responses about bonding social capital in more detail. Those who believe in reciprocity (“help each other” and “help others”) (agree and weakly agree) account for 31%–47%, which is larger than any other two aspects of bonding social capital. Overall, we observe that people's views did not change much as a consequence of the onset of the pandemic.

### 2.5 Trust in government (linking social capital)

The Wave 6 survey asked respondents about changes in their trust toward (1) the central government (“government trust”), (2) local government (“local government trust”), and (3) the medical system (“medical system trust”), compared to 2019 (the pre-pandemic period), which measure linking social capital (i.e., the vertical connections to the political center). Respondents were asked to select one of the following options regarding their change in trust in these three dimensions: “large increase (=5),” “increase (=4),” “no change (=3),” “decrease (=2),” or “large decrease (=1).” We note that the vaccine was licensed by the Ministry of Health, Labor and Welfare and thus trust in the medical system would implicitly include trust in this Central Government Ministry, which is charged with regulating the medical system.^13^

### 2.6 Health conditions and other health indicators

The survey asked about respondents’ existing illnesses (“existing illness”), COVID-19 infection (“own infection”), and mental health during the pandemic (“mental illness”), as well as any family infections of COVID-19 (“family infection”). These variables are recorded as binary variables (1 if these items were recorded). In addition, the survey asked respondents about their fear of COVID-19 infection (“fear infection”). Respondents were asked to choose from the following option: "always (=4)," "often (=3)," "sometimes (=2)," "a little bit (=1)," or "not at all (=0)." Additionally, the survey inquired about side effects experienced after previous vaccinations, such as fever (defined as higher than 37.5 °C), headache, pain, fatigue, other symptoms, or no symptoms. We constructed a dummy variable for side effects (“side effect”): no side effects (=0) and some/any side effects (=1).^14^

Finally, each wave also included the K6 questionnaire which measures six aspects of mental health. K6 is a mental health screening tool designed to monitor psychological distress, as proposed by Kessler et al. (2003).^15^ A higher K6 score indicates worse mental health. By comparing responses from waves 5 and 6, we construct a variable for changes in each respondent's mental health condition (“change K6”).

### 2.7 Other questions

Teleworking may reduce the incentive to vaccinate by eliminating the need for commuting, whereas the intention to commute and not work remotely could encourage vaccination. We include a telework binary indicator for whether a respondent engaged in teleworking at least one day during the fourth week of January 2022 (“teleworking”) in wave 6. During the COVID-19 pandemic, people were strongly encouraged to work from home, but some occupations and work environments were not suitable for teleworking (Okubo, 2022).

Additionally, some teleworkers experienced lower productivity and reduced job performance. As a result, some people were eager to return to their place of work, as they did before the pandemic, which may have increased their motivation to get vaccinated. In wave 4, the survey asked respondents about their preferences for commuting and teleworking in the post-COVID era. First, respondents were asked to select their preferred work style after the pandemic from the following options: (1) want to commute every day, (2) want to commute 3–4 days per week, (3) want to split time equally between commuting and teleworking, (4) want to telework 3–4 days per week, or (5) want to telework every day. Higher values indicate a greater desire to telework (“willingness to telework”).

Respondents who preferred teleworking for more than one day per week were then asked a follow-up question: if your employer offered additional salary to persuade you to commute every day after the pandemic, how much additional salary would you expect? The question was framed with a reference to a monthly salary of 400,000 yen.^16^ We construct a variable termed “money for commuting”.

Wave 6 also asked about changes in respondents’ daily lives since the previous survey wave (“life change” variables). This part included questions about working hours, time devoted to housekeeping, sleep, and leisure, income, expenditure, workload, job satisfaction, and life happiness.^17^ Respondents were asked to choose from the following: largely decrease, decrease, no change, increase, or largely increase.

## 3 Social capital, hypotheses, and estimands

Our main interest is bonding social capital. This term is subject to numerous interpretations; however we categorize three aspects, defined as (1) trust in other people, (2) willingness and belief in mutual help defined as reciprocity and (3) disposition to cooperate and contribute to one's community. These three non-exclusive aspects of bonding social capital may affect vaccination uptake in different ways.

Regarding Trust, those who have a high level of trust in others in their community tend to rely on them more. *Ceteris paribus*, they may also be more likely to "free ride" when it comes to vaccination, expecting herd immunity without getting vaccinated themselves. Therefore, their attitude toward vaccination may not involve protecting themselves, displaying a lack of interest in vaccination. They may believe they do not need to get vaccinated because someone else will, and herd immunity can still be achieved without their participation. On the other hand, because they are inclined to trust other people, they may also trust other people's views and information and therefore can be more easily convinced to vaccinate. Consequently, unlike the measures of ‘reciprocity’ and ‘cooperation’ discussed below, we do not have a clear hypothesis as to the direction of correlation between measures of ‘trust in others’ and the vaccination decision. The estimand we seek to identify in this case is therefore the statistical association between mutual trust (trust in others) and the vaccination decision, and shed some possible light on the nature of this association by examining the perceptions that underlie it.

As for ‘reciprocity’; the concept includes an aspect of altruism. Those who have strong reciprocity tend to view their own actions creating positive externalities for other people. They believe that mutual assistance is crucial, and that everyone getting vaccinated is an effective strategy. Thus, their perspective on vaccination is that it protects them while also protecting others. Since they are positively inclined toward vaccination and believe everyone should receive it, they think it is necessary for themselves to be vaccinated in order to help others (and they anticipate that others will reciprocate). *Ceteris paribus*, because of their belief in reciprocity, they are unlikely to stop seeking to vaccinate due to external factors, nor would they change their minds and start vaccination if they initially avoided it as their original decision most likely arose out of disbelief in the efficacy of the vaccine.

Lastly, regarding ‘cooperation’, in Japan, as in many other modern societies, many people commute to other regions for work and are less attached to their own communities. For this reason, they may not have strong incentive for vaccination. However, their preference is to cooperate, especially if they believe others will reciprocate, so they do not view the time and effort required for vaccination as too burdensome (cooperation justifies it). Accordingly, they view vaccinations as necessary to support the community as a whole. We therefore hypothesize that both cooperation and reciprocity are positively linked with the willingness to vaccinate, as examined not by the stated preferences of respondents but by their actions (their ‘revealed preferences’).

To summarize, our estimands are the statistical relationships between the three measures of bonding social capital and vaccinations preferences, while in the follow-up work we aim to identify how these beliefs shape those preferences and determine them. We would have liked to search for estimands that could be interpreted causally, but unfortunately the available data does not permit this. Ultimately, our estimates cannot be causally interpreted without additional assumptions about missing variables and other potential correlates of social capital and vaccination decisions.

## 4 Estimation method

### 4.1 Objective behavior and subjective opinion about the vaccine

We first investigate how social capital affects vaccination behavior and opinion about the vaccine. Using ordered logit, we regress

[1] $$prob\left(Y_{i}\right)=\phi \left({\beta X}_{i}+{\gamma Z}_{i}+\varepsilon_{i}\right)$$

where for the dependent variable ($Y_{i}$) is “${vacination}_{i}$” as the vaccination behavior indicator (0–5) that denotes the respondent's commitment to the vaccination campaign (from zero set as no intention to vaccinate to 5 denoting the respondent had already received the third dose), as discussed in section 2-2. $X_{i}$ is the set of variables we are focused on as determinants of the decision to vaccinate. These include measures of bonding social capital, trust in government (linking social capital), perceived fear of infection, own and family infection history, own mental health due to the pandemic, and concurrent illnesses. $Z_{i}$ is a vector of demographic and personal traits (gender, age, income, education, ICT skill, and teleworking), including occupation and firm-size effects.

We then re-specify the dependent variable ($Y_{i}$) as “${perception}_{i}$” where this is a vector of measures about the respondent's perception of the vaccine (1 = agree, 0 = neutral, and −1 = disagree). This variable includes above mentioned 10 questions about their views of the vaccination campaign, i.e. self-protection to no need of vaccination (section 2.3).

Finally, we attempt to combine these two investigations and estimate whether subjective views about the vaccine are at all related to the vaccination decisions that respondents actually make. Using the ordered logit estimation, we thus estimate the following specification:

[2] $$prob\left({vacination}_{i}\right)=\phi \left({\delta \;perception}_{i}+{\beta X}_{i}+{\gamma Z}_{i}+\varepsilon_{i}\right)$$

### 4.2 Changing views about the vaccination

As of wave 5 (September 2021), the maximum recommended vaccination regime was two doses, while between then and wave 6 (February 2022), the third dose was introduced and recommended. During this interval, it is possible that some people changed their mind on vaccination.

Using the information from waves 5 and 6, we also investigated the change of views on vaccination for two groups: (1) those that were previously not willing to vaccinate (in wave 5), but some of them have changed their views and decided to vaccinate by wave 6, and (2) those that have already taken at least one dose of the vaccine before wave 5, but have not taken any additional doses since then. The former one is to express a binary indicator for starting to vaccinate (“Start”) and the latter one is a binary indicator for stopping to vaccinate (“Stop”). For these, we estimate a probit model. For this model, we also add a set of life change variables that denote life changes (changes of working hours, housekeeping hours, sleeping hours, leisure hours, income, amount of task, job satisfaction, life happiness, expenditure, and mental health) experienced by the respondents between wave 5 and wave 6, which may explain the change in the respondents’ choices (see Section 2-7). In addition, we add the number of new infections at municipality level as of September 2021.

[3] $$prob\left({change}_{i}\right)=\phi \left(\alpha +{\beta X}_{i}+{\gamma Z}_{i}+\delta {Life}_{i}+\varepsilon_{i}\right)$$

where “${change}_{i}$” denotes either a binary indicator for starting vaccination (“start”) or stopping vaccination (“stop”) as mentioned above.

## 5 Results

We first examine the estimations result from a model that explains the decision to vaccinate (see equation (1)) in Table 2. Table 2 reports the marginal effect of the ordered logit model at vaccination = 5 (received the third dose of vaccine). Appendix Figure (Fig. A1) reports marginal effects for social capital variables (Trust, Reciprocity, and Regional Coop) at all points of vaccination choices (=0,1,2,3,4,5). For instance, marginal effects of Trust are obtained for the probability for choosing 0 (1, 2, 3, 4, 5). The specifications include first the ones including only the benchmark social capital variables and socio-demographic controls (column 1). It then adds occupation, firm-size, and employment status effects (column 2), a set of trust measures (column 3), a set of health measures (column 4), and additional controls for the willingness to work remotely and hypothetical compensation for on-site work in columns 5 and 6. We note that these last two questions include many missing responses which reduces the sample by about a third, because the willingness for teleworking was asked in wave 4 (see section 2-1) and some respondents did not participate in that survey.

**Table 2:** Ordered logit models – vaccination behavior.

VARIABLES	(1)	(2)	(3)	(4)	(5)	(6)
	vaccination	vaccination	vaccination	vaccination	vaccination	vaccination
trust_people	0.003	0.004	0.005∗∗	0.007∗∗∗	0.008∗∗	0.008∗∗
	(1.260)	(1.585)	(2.012)	(2.749)	(2.519)	(2.483)
reciprocity	0.017∗∗∗	0.015∗∗∗	0.014∗∗∗	0.010∗∗∗	0.009∗∗∗	0.009∗∗
	(6.175)	(5.778)	(5.222)	(4.094)	(2.672)	(2.508)
regional coop	−0.003	−0.002	−0.002	−0.003	−0.003	−0.004
	(-1.063)	(-0.785)	(-0.957)	(-1.201)	(-0.851)	(-1.165)
government_trust			−0.002	0.000	0.002	0.004
			(-0.607)	(0.0432)	(0.463)	(0.877)
local_government_trust			0.001	0.002	−0.000	−0.001
			(0.291)	(0.499)	(-0.0853)	(-0.285)
medical_system_trust			0.007∗∗∗	0.006∗∗	0.008∗∗	0.009∗∗
			(2.714)	(2.241)	(2.469)	(2.527)
exsting_illness				0.007	0.006	0.012∗∗
				(1.408)	(1.104)	(2.086)
mental_illness				−0.029∗∗∗	−0.034∗∗∗	−0.038∗∗∗
				(-3.644)	(-3.483)	(-3.517)
own_Covid_infection				−0.036∗∗∗	−0.025∗∗∗	−0.022∗∗∗
				(-6.481)	(-3.807)	(-3.099)
family_Covid_infection				−0.002	−0.008	−0.002
				(-0.275)	(-0.826)	(-0.225)
ICT skill	0.002	0.002	0.003	0.004∗	0.006∗∗	0.006∗∗
	(1.094)	(1.032)	(1.198)	(1.692)	(2.260)	(2.096)
teleworking			0.002	0.004	0.003	0.000
			(0.472)	(1.037)	(0.577)	(0.0699)
sex	0.013∗∗∗	0.004	0.004	0.003	0.005	0.005
	(3.808)	(1.134)	(1.007)	(1.092)	(1.204)	(1.111)
age	0.012∗∗∗	0.013∗∗∗	0.013∗∗∗	0.012∗∗∗	0.012∗∗∗	0.012∗∗∗
	(17.55)	(18.12)	(17.77)	(16.65)	(13.44)	(12.70)
education	0.007∗∗∗	0.003∗∗	0.004∗∗	0.003∗∗	0.006∗∗∗	0.008∗∗∗
	(4.097)	(2.014)	(2.158)	(2.043)	(3.086)	(3.423)
income	−0.000	−0.001∗∗∗	−0.001∗∗∗	−0.001∗∗	−0.001	−0.001
	(-0.477)	(-2.964)	(-3.120)	(-2.171)	(-1.393)	(-1.141)
fear_infection			0.012∗∗∗	0.013∗∗∗	0.012∗∗∗	0.013∗∗∗
			(9.748)	(10.42)	(7.741)	(8.009)
side effect			0.049∗∗∗	0.050∗∗∗	0.050∗∗∗	0.050∗∗∗
			(14.16)	(14.21)	(12.12)	(11.44)
Willingness of Teleworking					−0.006∗∗∗	
					(-4.344)	
Money receive for commuting						−0.003∗∗∗
						(-3.790)

Observations	9341	9341	9341	9341	6427	5663

Pseudo R-2	0.028	0.061	0.073	0.079	0.081	0.082
Wald chi2	533.490	1006.660	1269.720	1443.260	1269.180	1144.880
Log pseudolikelihood	−10133.6	−9789.6	−9663.3	−9600.2	−6443.7	−5690.7

Occupation, Firm, and Employment status effects	No	Yes	Yes	Yes	Yes	Yes

Note: Marginal effect for outcome (vaccination = 5). Robust z-statistics in parentheses. ∗∗∗p < 0.01, ∗∗p < 0.05, ∗p < 0.1.

Overall, the picture that emerges from the regressions in Table 2 appears to be quite consistent. Bonding social capital matters, in particular, the trust that people have in other people in their community (trust people), and their willingness and belief in the need for mutual help (reciprocity). The more ill-defined dimension of bonding social capital – belief in cooperation within the community (cooperation)– does not seem to be associated with the vaccination decision at all.

Those respondents who believe in mutual reciprocity and trust in other community members tend to be more supportive of vaccination and vaccinate themselves accordingly. The marginal effects for reciprocity are larger than for trust in most specifications, indicating belief in reciprocity plausibly plays a more central role in promoting vaccination. As discussed in Section 3, belief in reciprocity involves support for an altruistic motive, and thus could be a driver for vaccination. On the other hand, since communities are now less cohesive (or geographically well-defined) in many places, it is possible that those who believe in a cooperative framework still do not think vaccination is beneficial to those who are proximate to them.

The variables measuring trust in central and local government (linking social capital) are consistently not statistically significant; as noted earlier, that may be because, in Japan, the actions of government during the pandemic were not as controversial as in many other countries. Maybe not surprisingly, however, the measure of trust in the health system is statistically significant and positive, and with the same magnitude of association as the bonding social capital measures of trust in others and reciprocity.

We also note that the magnitude of this association between trust-in-people and reciprocity, and the vaccination decision, is less pronounced once we control for the full set of covariates, but it remains statistically significant even in columns 5–6 when all controls are included (and the sample is reduced). Furthermore, there are no noticeable differences in terms of the size of these coefficients once the full covariate list is included.

Perhaps less trivially, we also find that people who are facing mental health challenges are associated with less willingness to receive the vaccine. From a public health perspective, this finding may be important, as there may be interventions that can assist people with mental health and thus increase their willingness to vaccinate, but exploring these possibilities is beyond what is feasible with the survey data we have. We also note that respondents who have been infected by COVID-19 previously, are less likely to vaccinate. This is less surprising, as it was widely known at the time that previous infection from the virus allows for at least a partial immunity from consequent infections (though that has changed later as new variants of the virus emerged).

Some other variables that are included in the specifications as controls – age, education – were positively associated with vaccinations, as was the fear of infection. These results are all intuitive and hardly need explaining given the age pattern of vulnerability to the virus. More surprisingly, the experience of side effects is associated with an increase in vaccination. Finally, in columns 5 and 6 we observe that people who viewed remote working more favourably were less willing to vaccinate, as were people who thought they would require higher compensation to be convinced to ‘return to the office’. In Japan, many workers commute to work in very crowded public transportations for long commutes (often involving more than one mode of transport). It is well recognized in Japan that one of the main infection hotspots is these congested commuting trains and buses – e.g., during the annual flu season. As such, it is not surprising that those who are more willing to tele-work, or who can do so more easily, are less keen on vaccinations.

In the supplemental information (Appendix Table A2 and Fig. A1), we detail the results we obtained for the occupation, firm size, and employment-status effects for Table 3 estimates (columns 2–4) and the average marginal effects for column 4, respectively. These find that people who work in larger firms or in the public sector are more likely to vaccinate, as are people who work in the health and social service sectors. There were several other sectors that were associated more weakly with a lower propensity to vaccinate. The self-employed were also less likely to vaccinate.

**Table 3:** Ordered logit models – vaccination perceptions.

VARIABLES	(1)	(2)	(3)	(4)	(5)	(6)	(7)	(8)	(9)	(10)
	self protection	protect others	safety concern	side effects concern	time concern	reservation system	no infection	no COVID	mask enough	no need
trust_people	−0.009	−0.010	−0.033∗∗∗	−0.072∗∗∗	−0.001	−0.030∗∗∗	0.015∗∗∗	0.017∗∗∗	0.012∗∗∗	0.014∗∗∗
	(-1.049)	(-1.091)	(-4.781)	(-7.990)	(-0.314)	(-4.018)	(4.538)	(4.454)	(2.998)	(3.680)
reciprocity	0.100∗∗∗	0.110∗∗∗	0.001	0.074∗∗∗	−0.040∗∗∗	−0.010	−0.026∗∗∗	−0.032∗∗∗	−0.036∗∗∗	−0.043∗∗∗
	(11.32)	(12.34)	(0.0788)	(7.831)	(-9.747)	(-1.343)	(-7.556)	(-7.944)	(-8.986)	(-10.88)
regional coop	0.008	0.010	−0.009	0.010	−0.013∗∗∗	−0.017∗∗	−0.012∗∗∗	−0.014∗∗∗	−0.012∗∗∗	−0.012∗∗∗
	(0.941)	(1.090)	(-1.223)	(1.107)	(-3.523)	(-2.315)	(-3.650)	(-3.665)	(-3.103)	(-3.422)
government_trust	−0.0210∗∗	−0.021∗∗	−0.021∗∗	−0.051∗∗∗	0.018∗∗∗	−0.005	0.025∗∗∗	0.018∗∗∗	0.016∗∗∗	0.028∗∗∗
	(-1.963)	(-1.974)	(-2.394)	(-4.756)	(3.889)	(-0.608)	(6.036)	(3.823)	(3.401)	(5.945)
local_gov_trust	0.002	−0.001	−0.032∗∗∗	−0.038∗∗∗	0.001	−0.020∗	0.001	0.006	0.008	0.003
	(0.138)	(-0.0605)	(-3.177)	(-3.092)	(0.122)	(-1.914)	(0.139)	(1.137)	(1.526)	(0.508)
med_syst_trust	0.047∗∗∗	0.045∗∗∗	−0.022∗∗∗	−0.004	−0.022∗∗∗	−0.019∗∗	−0.029∗∗∗	−0.040∗∗∗	−0.039∗∗∗	−0.027∗∗∗
	(4.795)	(4.679)	(-2.769)	(-0.464)	(-5.210)	(-2.230)	(-7.730)	(-8.730)	(-8.958)	(-6.488)
illness	0.020	0.044∗∗∗	0.017	0.025	−0.034∗∗∗	−0.032∗∗	−0.035∗∗∗	−0.044∗∗∗	−0.036∗∗∗	−0.043∗∗∗
	(1.196)	(2.604)	(1.262)	(1.513)	(-4.397)	(-2.287)	(-5.357)	(-6.005)	(-4.954)	(-5.577)
mental_illness	−0.081∗∗∗	−0.116∗∗∗	−0.039∗	−0.020	0.031∗∗∗	0.055∗∗	0.029∗∗∗	0.026∗∗	0.012	0.032∗∗∗
	(-3.126)	(-4.579)	(-1.766)	(-0.803)	(2.629)	(2.471)	(2.635)	(2.115)	(1.063)	(2.884)
own_infection	−0.157∗∗∗	−0.145∗∗∗	−0.034∗∗	−0.142∗∗∗	0.042∗∗∗	−0.029∗∗	0.055∗∗∗	0.044∗∗∗	0.047∗∗∗	0.070∗∗∗
	(-10.34)	(-9.417)	(-2.510)	(-9.055)	(6.136)	(-2.350)	(8.354)	(5.994)	(6.523)	(10.69)
family_infection	0.079∗∗∗	0.056∗∗	0.024	0.000	0.025∗∗	0.000	−0.002	0.025∗∗	0.025∗∗	0.006
	(3.128)	(2.215)	(1.139)	(-0.000832)	(2.300)	(0.0118)	(-0.219)	(2.137)	(2.217)	(0.518)
ICT skill	0.031∗∗∗	0.028∗∗∗	0.014∗∗	0.028∗∗∗	−0.006∗∗	0.009∗	−0.004∗	0.002	−0.000	−0.004∗
	(4.827)	(4.329)	(2.551)	(4.352)	(-2.205)	(1.708)	(-1.830)	(0.567)	(-0.145)	(-1.802)
teleworking	0.080∗∗∗	0.071∗∗∗	0.023∗∗	0.048∗∗∗	−0.000	0.047∗∗∗	0.008	0.007	0.010∗	0.007
	(5.667)	(5.138)	(2.033)	(3.443)	(-0.00367)	(4.108)	(1.465)	(1.195)	(1.757)	(1.289)
sex	0.037∗∗∗	0.036∗∗∗	0.049∗∗∗	0.114∗∗∗	−0.001	0.022∗∗	−0.010∗∗	−0.015∗∗∗	−0.015∗∗∗	−0.010∗∗
	(3.498)	(3.398)	(5.627)	(10.40)	(-0.173)	(2.378)	(-2.255)	(-3.122)	(-3.080)	(-2.185)
age	0.027∗∗∗	0.027∗∗∗	−0.010∗∗∗	−0.013∗∗∗	−0.013∗∗∗	−0.017∗∗∗	−0.004∗∗∗	−0.009∗∗∗	−0.011∗∗∗	−0.008∗∗∗
	(14.82)	(14.35)	(-5.906)	(-6.536)	(-14.86)	(-10.36)	(-5.445)	(-9.915)	(-12.60)	(-10.01)
education	0.009∗	0.010∗	−0.009∗∗	−0.002	−0.004	−0.000	−0.002	−0.001	−0.004∗	−0.004∗∗
	(1.792)	(1.938)	(-2.237)	(-0.297)	(-1.588)	(-0.0411)	(-1.274)	(-0.811)	(-1.738)	(-2.069)
income	−0.005∗∗∗	−0.003∗∗	−0.000	−0.002∗	0.002∗∗∗	0.002∗∗	0.002∗∗∗	0.002∗∗∗	0.001∗	0.002∗∗∗
	(-3.955)	(-2.386)	(-0.263)	(-1.767)	(3.980)	(2.168)	(3.597)	(3.289)	(1.705)	(4.116)


Pseudo R-2	0.0705	0.0716	0.0239	0.045	0.0722	0.0266	0.0397	0.0454	0.0534	0.0642
Wald chi2	1007.05	1055.84	397.95	795.22	1152.89	484.96	629.93	769.11	846.73	992.24
loglikelihood	−7528.65	−7735.741	−9059.053	−8917.9	−8227.779	−9912.444	−8463.079	−8703.436	−8559.672	−8024.185

Note: Marginal effects. Robust z-statistics in parentheses. ∗∗∗p < 0.01, ∗∗p < 0.05, ∗p < 0.1. All regressions include occupation, firm size, and employment status effects. They all include the full sample size (9,341).

In the next set of specifications, in Table 3, we present estimations that examine the association between respondents’ perceptions about the vaccinations, and the benefits and needs to vaccinate (section 2, 3) and the bonding (and linking) social capital measures we described above. These views about vaccinations can be divided into three parts: (1) The perceived benefits of vaccinations (e.g., vaccine provides protection from infection for family members); (2) The perceived non-monetary costs of vaccinations (e.g., the side effects of the vaccine and time loss from messy reservation system); and (3) reasons why vaccinations are (un)necessary (e.g., masks provide sufficient protection, deny COVID-19 is real).

As shown in Table 3, the first type of views (benefits – columns 1–2) is associated positively and significantly with reciprocity, but not with the other measures of bonding social capital (inter-personal trust and cooperation). People who place more importance on reciprocity tend to view vaccination as beneficial, believing it protects them and others. The second type (costs – columns 3–6) the observed pattern of association is less clear and is more difficult to generalize. Each specific perception about the costs of getting vaccinated seems to be associated differently with social capital. Interestingly, it is ‘trust in others’ that is consistently negative (though not always with statistical significance), and the other measures are inconsistently signed. For the third type of views that are doubting the necessity of vaccination (columns 7–10), the emerging association is consistent. It is positive with the ‘trust in others’ variable, so that people who trust other people see these skeptical views as more legitimate. This variable has the opposite sign in columns 7–10 compared to the results described in columns 1–6. Meanwhile, both reciprocity and regional cooperation are consistently negative and statistically significant. Those who believe in reciprocity and cooperation identify less with these doubting views about the need vaccinate.

As discussed in the hypothesis section (section 3), individuals with a high level of trust in others may be more willing to rely on others and therefore “free ride” when it comes to vaccination, expecting the community to achieve herd immunity without them. Their rationale is that they do not need to be vaccinated because others will, allowing herd immunity to be achieved without their participation.

On the other hand, reciprocity involves an element of altruism. Individuals who value reciprocity tend to view their own vaccination as generating positive externalities for others. From their perspective, vaccination protects both themselves and others. Because they believe vaccination is a collective responsibility, they are more likely to be concerned about its safety and potential side effects. They see vaccination as a necessary action not only for their own benefit but also to help others. Regarding cooperation, modern society has seen the decline of many local communities. However, their view of vaccination may be shaped by the belief that the time and effort required for it are not overly burdensome. They see vaccination as necessary to support the community as a whole.

The main insight we conclude from the results described in Table 3 is that the association between social capital and vaccination behavior is mediated by the ways people view the vaccination, and those views are inconsistently related to social capital. For example, people who have some types of bonding social capital can believe less in the arguments that suggest the vaccine is unnecessary, and also believe the arguments that it is beneficial, but have a less clear views about the associated costs of undertaking vaccination. Table 3 reports the marginal effect of ordered logit model at each opinion = 1 (agree). Appendix Fig. A2 reports marginal effects for social capital variables (Trust, Reciprocity, and Regional Coop) at all points of opinions (=-1,0,1). For instance, marginal effects of Trust are obtained for the probability for choosing −1,0,1.

In further investigation, presented in Table 4, we examine the association between these ten views (about the benefits, costs and necessity of the vaccine) and the decision to vaccinate (equation (2)) while also including the bonding social capital measures. Table 4 reports marginal effect of ordered logit model at vaccination = 5 (received the third dose of vaccine). Appendix Fig. A3 reports marginal effects for social capital variables (Trust, Reciprocity, and Cooperation) at all points of vaccination choices (=0,1,2,3,4,5). The ‘benefit’ views are positively associated with vaccination behavior, the ‘cost’ views are negatively associated with vaccination behavior, and the neutral ‘unnecessary’ views do not show a consistent pattern. We note that some basic variables, socio-demographic controls (age, income, sex, ICT skill, and education), a set of government trust measures (linking social capital), and a set of health measure such as in column 4 of Table 3 are all included in the estimation but not reported.

**Table 4:** Ordered Logit model – Vaccination Behavior and Perceptions.

VARIABLES	vaccination
self-protection	0.060∗∗∗
	(15.44)
protect others	0.037∗∗∗
	(10.14)
not safe	−0.004
	(-1.414)
side effects	−0.012∗∗∗
	(-4.474)
no time	−0.009∗∗∗
	(-3.620)
no reservations	−0.014∗∗∗
	(-5.105)
no infection	−0.003
	(-1.441)
no covid	0.014∗∗∗
	(4.954)
mask	−0.008∗∗
	(-2.362)
no need	−0.036∗∗∗
	(-10.58)
trust_people	0.005∗∗
	(2.098)
reciprocity	−0.004
	(-1.592)
Regional coop	−0.004
	(-1.627)
Pseudo R-2	0.191
Wald chi2	3986.59
loglikelihood	−8433.774

Note: Marginal effect for outcome (vaccination = 5). Robust z-statistics in parentheses. ∗∗∗p < 0.01, ∗∗p < 0.05, ∗p < 0.1. Regression includes occupation, firm size, and employment status effects and the other control variables included in column 4 Table 2. The full sample size (9,341) is used.

Table 5 looks at the reasons people change their plans with respect to vaccination (equation (3)). Between wave 5 and wave 6, some respondents decide to finally start to vaccinate (Column 1), and some decide to stop their vaccinations (Column 2). For these estimations, we also use several questions from the Wave 6 survey that asked people about changes they have experienced recently (since they were surveyed in Wave 5). For these change variables, in Column 1, we see that people that decide to start vaccinating (to take their first dose, when the third dose is already available) are people who increased their working hours and their higher income, but that their life happiness has decreased between the two survey waves. In this case, people with a higher degree of trust in other people were more likely to change their mind and start vaccinating than those who decided to maintain their earlier no-vaccine decision. There is no apparent difference between these two groups in terms of their beliefs in reciprocity and cooperation.

**Table 5:** Probit models – changing vaccination behavior.

VARIABLES	(1)	(2)
	Start	Stop
Δ working hours	−0.087∗	−0.008
	(-1.851)	(-0.872)
Δ housekeeping hours	−0.016	0.014
	(-0.350)	(1.601)
Δ sleeping hours	−0.026	−0.013
	(-0.551)	(-1.472)
Δ leisure hours	0.049	0.007
	(1.172)	(0.892)
Δ Income	0.090∗∗	−0.001
	(2.276)	(-0.164)
Δ amount of tasks	0.056	−0.008
	(1.195)	(-1.036)
Δ job satisfaction	−0.018	0.023∗∗∗
	(-0.443)	(2.607)
Δ life happiness	−0.092∗∗	−0.002
	(-2.166)	(-0.251)
Δ expenditure	−0.029	−0.007
	(-0.898)	(-0.955)
Δ mental health	0.030	−0.001
	(0.811)	(-0.0617)
Δ K6	−0.026	0.003
	(-1.524)	(0.832)
trust_people	0.082∗∗	−0.018∗∗
	(2.549)	(-2.358)
reciprocity	−0.047	−0.024∗∗∗
	(-1.261)	(-3.113)
Regional coop	0.035	0.004
	(1.036)	(0.596)
Side effect		−0.064∗∗∗
		(-4.402)
covid	2.101∗	−0.068
	(1.763)	(-0.219)
Observations	524	3511
R-sq	0.130	0.120
LR-chi	89	231.94
Log likelihood	−298.100	−848.363

Note: Marginal effect for outcome (vaccination = 5). Robust z-statistics in parentheses. ∗∗∗p < 0.01, ∗∗p < 0.05, ∗p < 0.1. Regression includes occupation, firm size, and employment status effects and the other control variables included in column 4 Table 2. The full sample size (9,341) is used.

For the opposite change, people who decide to stop vaccinating, having already received at least one dose of the vaccine, the results are different. They are more satisfied with their work than those who chose to continue vaccinating. This group of ‘stoppers’ also trust less in other people and believes less in their community's support of reciprocity. Belief in cooperation is not statically significant as a correlate of the decision to stop.

## 6 Discussions and conclusions

Our results are informing the literature on vaccination hesitancy. Previous studies found a strong preference for vaccines that were developed and approved locally – or alternatively less willingness to rely on foreign vaccines (Kawata & Nakabayashi, 2021; Kreps et al., 2020; Motta, 2021; Schwarzinger et al., 2021; Sun et al., 2020). This is not our focus, but the bonding social capital we focus on may be related to the perception of differences between domestic and foreign vaccines. In our results, we find that higher bonding social capital can reduce vaccination hesitancy. However, the results are more nuanced as bonding social capital is a broad concept that encompasses many separate concepts. We focus on inter-personal trust, and on beliefs about reciprocity and cooperation within the community.

Overall, our results indicate that reciprocity is beneficial to vaccination take-up. Reciprocity, as a concept, involves a degree of altruism. Thus, those who have a strong belief in reciprocity within the community tend to have stronger incentives to take the vaccination and have perceptions of its benefits to society. Achieving herd immunity is an important public health priority. Once a large majority has taken a vaccine, this establishes herd immunity against a disease, but, ironically, herd immunity permits people to free-ride and not vaccinate. Preventing such free riding is a serious policy challenge. Kawata and Nakabayashi (2021) showed that mandated vaccination would decrease vaccine hesitancy and concluded that mandated vaccination could be a solution to contain vaccine hesitancy motivated by free riding. However, our results indicate that social capital, in particular reciprocity is potentially an alternative solution. Those who believe in reciprocity tend to consider the positive externality of their own vaccination decisions and are thus more willing to take the vaccinations.

What can we conclude from our results? We mostly found support, in the Japanese data, for the view that social capital matters for the vaccination decision; and that it matters even once we control for institutional trust – linking social capital (especially trust in the medical system). However, this general association between trust in other community members, trust in the willingness of community members to engage in reciprocal assistance, and trust in the more general willingness of the community to support individuals, were all associated differently with the vaccination decision, and with the views about vaccinations (which we divided into views about the benefits, the costs, and the necessity of vaccination).

In Japan, trust in government, and in government actions associated with the pandemic lockdowns, were not associated with the decision to vaccinate. However, trust in the other members of the community remains a statistically important determinant of vaccination decision even once trust in the medical system, local, and central government was all accounted for. From a policy perspective, this suggests that Japan's case shows again the relevance of trust in government, but also shows that intra-community trust (i.e., bonding social capital), is important in its various hues, even in contexts when governmental trust is not a significant concern.

Overall, we can conclude that any policy aims at facing the public health challenges associated with a new vaccination campaign should also be considering the various facets of bonding social capital. This insight, therefore, would support the view that for this reason, and indeed for many others, social capital should be deliberately encouraged, supported, and maintained, so that policymakers can adequately use it when a crisis requires it.

## CRediT authorship contribution statement

**Toshihiro Okubo:** Writing – original draft, Validation, Software, Project administration, Methodology, Investigation, Funding acquisition, Formal analysis, Data curation, Conceptualization. **Ilan Noy:** Writing – review & editing, Writing – original draft, Validation, Supervision, Investigation, Conceptualization.

## Ethical statement

We declare that we follow all ethical guidelines for the paper “**Vaccination Decisions and Social Capital in Japan**” by Okubo and Noy.

## Declaration of competing interest statement

We declare that we have no relevant or material financial interests that relate to the research described in the paper “**Vaccination Decisions and Social Capital in Japan**” by Okubo and Noy.

## Data Availability

The data that has been used is confidential.

## Footnotes

1 https://www.kantei.go.jp/jp/headline/kansensho/vaccine_supply.html.

2 Specific groups were more hesitant. Takahashi et al. (2022) find that 51% of pregnant women and 32% of mothers of young children were vaccine hesitant.

3 Possibly counter-intuitively, vaccinated people were also found to adopt preventive behaviors, such as mask wearing and improved hand hygiene, after vaccination (Yamamura et al., 2024).

4 This even led to situations in which the opposition to vaccines was mostly from the Right (e.g., in the US or Canada) or from the Left (e.g., in Israel) as the government was from the ‘other side of the aisle.’

5 https://www.japantimes.co.jp/opinion/2020/04/14/commentary/japan-commentary/coronavirus-japans-constitution/. https://www.reuters.com/article/world/lockdown-japan-style-pressure-to-conform-not-penalties-for-non-compliance-idUSKBN21P0EU/.

6 In response to the pandemic, there were some who called to amend the Constitution to include emergency provisions that would allow the government to enforce control measures in case of national emergencies. There are others who still strongly oppose such proposals.

7 As was done, for example, in Allington et al. (2023) in the UK, Blamey and Noy (2024) in New Zealand, Colegrove et al. (2022) in the US, Falcone et al. (2022) in Italy, Jang (2022) in Korea, Mankell and Abdelzadeh (2023) in Sweden, and Pagliaro et al. (2021) in cross-country analysis.

8 See https://nira.or.jp/paper/data/2022/26.html for the data link

9 The sample sizes in the first, second, third, fourth, fifth, sixth, and seventh waves were 10,516, 12,138, 10,523, 9,796, 10,644, 10,113, and 10,595, respectively. The survey was conducted on a website constructed by Nikkei Research Co. The survey takes a stratified random sampling strategy. Japan is stratified into five regions by regional classification and six age groups for each gender (12 age-gender groups per region). The number of samples for 60 region–age-gender groups was determined by population ratio. The Labor Force Survey (Ministry of Internal Affairs and Telecommunication) was employed as the sampling unit. The survey intended to construct the panel structure and thus keep the same respondents over waves as much as possible. Some respondents repeatedly participated in the survey, and some did not. New respondents were added to fill in the allocated number of samples in each unit.

10 The number of daily new infections per population (i.e. infection rate) is as of the first day of the month when the survey was conducted. We collected the numbers of daily new infections at each municipality from each municipality's website. In rural areas, the number is not available at the municipality level but available at the health-center level jointly handled by nearby municipalities. The number in the rural area is derived from the health-center level number divided by population of the municipalities covered.

11 Most famously, Putnam et al. (1993) argued that the reason why northern Italy developed faster than southern Italy is its abundant endowments of social capital, which, in their framing refers to trust of others, reciprocity, and local cooperation. In that case, they argued, social capital enhanced the efficient organizing of society.

12 We note that the survey asked social capital as of pre-COVID, as well at the time of the survey; our paper uses the latter. Our social capital framework is very similar to Yodo (2018) who defined the three dimensions of social capital similarly. Other similar frameworks applied in the Japanese context can be found in Kawachi et al. (1999) and Hamano et al. (2009).

13 Thus, the measured trust in the medical system may also include the effects of trust in the government, and as such lead to an underestimation of the effects of trust in the government. Fukai et al. (2023) found a deep divide of trust in vaccine licensing by the Ministry of Health, Labour and Welfare between respondents who took the first dose of COVID-19 vaccine and those who chose not to take any.

14 Respondents who had never been vaccinated were assigned a value of zero (no side effects).

15 The K6 has been translated into multiple languages, including Japanese. We use the Japanese version from Furukawa et al. (2008).

16 400,000 yen is used as reference in the survey question. Average monthly income for regular workers in Japan is 369,239 yen as of January 2023 (Monthly Labour Survey by the Ministry of Health, Labour and Welfare). At the time the question was asked, this was about US$ 3000.

17 During the pandemic, the Japanese people changed time allocation. See Okubo (2025).
