# Supplementary material for: Vaccination decisions and social capital in Japan
Source: SSM Popul Health. 2025 Feb 26;30:101769. doi: 10.1016/j.ssmph.2025.101769 (PMC11925093; doi:10.1016/j.ssmph.2025.101769)
Supplement: Multimedia component 1 [file mmc1.docx]

**Appendix Section for “Vaccination Decisions and Social Capital in Japan”**

**(ONLINE SUPPLEMENT)**

January 2025

**Abstract**: The COVID-19 vaccines played a pivotal role in safeguarding many people. Yet, vaccine hesitancy remained a significant barrier to increasing coverage rates, as many high-income countries faced prolonged vaccine refusal campaigns. In Japan, vaccine doses were administered under a reservation system accessible via a website and by phone. Achieving a high vaccination coverage for a vaccine that was offered at no financial cost was surprisingly difficult in Japan as well. In many countries, vaccine hesitancy during the pandemic has been closely related to people's trust in their governments given governments’ controversial social distancing mandates. In Japan, lockdowns were voluntary, and vaccinations were also not mandated. As there were no significant political conflicts about the government’s policies, vaccination acceptance was influenced by more basic tenets, and we focus here on social capital, defined as cohesive links that enable a society to function effectively. Social capital, in this context, refers to community trust, collaboration, and engagement that create social bonds between individuals and society. Using a uniquely large survey, administered repeatedly through the years of the pandemic, we mostly find support, for the hypothesis that social capital matters for the vaccination decision; and that it matters even once we control for institutional trust (especially trust in the medical system). However, this general association between trust in other community members, belief in the willingness of community members to engage in reciprocal assistance, and belief in the more general willingness of the community to support individuals, were all associated differently with the vaccination decision, and with the views expressed about the vaccinations. From a policy perspective, this suggests that intra-community trust (i.e., bonding social capital), is important even in contexts when trust in governmental is not a significant concern.

Keywords: Vaccine, COVID-19, social capital, trust

**Appendix Table A1: Basic Statistics**

| stats | N | mean | min | max |
| --- | --- | --- | --- | --- |
| vaccination | 9585 | 3.445175 | 0 | 5 |
| self_protect | 9585 | 0.535733 | -1 | 1 |
| other_protect | 9585 | 0.502765 | -1 | 1 |
| safety concern | 9585 | 0.065519 | -1 | 1 |
| side_effect concern | 9585 | 0.341784 | -1 | 1 |
| time concern | 9585 | -0.42984 | -1 | 1 |
| reserve system | 9585 | -0.03036 | -1 | 1 |
| no_infection | 9585 | -0.3687 | -1 | 1 |
| no_covid | 9585 | -0.323 | -1 | 1 |
| mask is enough | 9585 | -0.37194 | -1 | 1 |
| no_need | 9585 | -0.47846 | -1 | 1 |
| trust_people | 10113 | 3.011174 | 1 | 5 |
| reciprocity | 10113 | 3.313359 | 1 | 5 |
| regional coop | 10113 | 3.15841 | 1 | 5 |
| government trust | 10113 | 2.764066 | 1 | 5 |
| local_government trust | 10113 | 2.833284 | 1 | 5 |
| medical system trust | 10113 | 2.981707 | 1 | 5 |
| existing_illness | 10113 | 0.123208 | 0 | 1 |
| mental_illness | 10113 | 0.056462 | 0 | 1 |
| own_covid infection | 10113 | 0.115495 | 0 | 1 |
| family COVID infection | 10113 | 0.051617 | 0 | 1 |
| stop | 6310 | 0.073376 | 0 | 1 |
| start | 1078 | 0.419295 | 0 | 1 |
| sex | 10113 | 1.448037 | 1 | 2 |
| age | 10113 | 7.938001 | 2 | 12 |
| education | 10113 | 3.292693 | 1 | 6 |
| income | 10113 | 5.384307 | 0.25 | 21.25 |
| ICT skill | 9830 | 1.331536 | 0 | 3 |
| teleworking | 10113 | 0.167408 | 0 | 1 |
| fear_infection | 10113 | 1.508356 | 0 | 4 |
| side effect | 10113 | 0.165332 | 0 | 1 |

**Appendix Table A2: Firm size and employment status effects in Table 2**

|  |  | 2 | 3 | 4 |
| --- | --- | --- | --- | --- |
| Firm size: reference 1-4 employees | |  |  |  |
|  | 5-29 employees | 0.00107 | 0.00165 | 0.00338 |
|  |  | (0.165) | (0.264) | (0.555) |
|  | 30-99 employees | 0.00830 | 0.00879 | 0.0128** |
|  |  | (1.210) | (1.324) | (1.961) |
|  | 100-499 employees | 0.00945 | 0.00914 | 0.0115* |
|  |  | (1.380) | (1.377) | (1.775) |
|  | More than 500 employees | 0.0114* | 0.0130** | 0.0153** |
|  |  | (1.714) | (2.002) | (2.417) |
|  | Government and public | 0.0136 | 0.0145 | 0.0161* |
|  |  | (1.470) | (1.592) | (1.804) |
| employment status: reference regular workers | | |  |  |
|  | non-regular | -0.00871** | -0.00796** | -0.00763* |
|  |  | (-2.134) | (-1.971) | (-1.907) |
|  | employers | -0.00386 | -0.00580 | -0.00558 |
|  |  | (-0.343) | (-0.551) | (-0.550) |
|  | self-employment, with employees | -0.0247*** | -0.0227*** | -0.0175* |
|  |  | (-2.762) | (-2.585) | (-1.919) |
|  | self-employment, no employees | -0.0320*** | -0.0293*** | -0.0287*** |
|  |  | (-4.827) | (-4.453) | (-4.376) |
|  | home work and family business | -0.0466*** | -0.0426*** | -0.0396*** |
|  |  | (-5.550) | (-4.904) | (-4.519) |
| Occupation: reference 1: adiministrative and managerial | | |  |  |
| 2 | Researchers | -0.0188* | -0.0158 | -0.0121 |
|  |  | (-1.791) | (-1.424) | (-1.072) |
| 3 | Agricultural engineers | -0.00517 | -0.00731 | -0.00658 |
|  |  | (-0.199) | (-0.296) | (-0.271) |
| 4 | Manufacturing engineers | -0.0120* | -0.0128* | -0.0129* |
|  |  | (-1.750) | (-1.866) | (-1.897) |
| 5 | Architects, civil engineers | -0.0191*** | -0.0215*** | -0.0180*** |
|  |  | (-2.790) | (-3.224) | (-2.613) |
| 6 | Data processing | -0.00939 | -0.00901 | -0.00902 |
|  |  | (-1.375) | (-1.296) | (-1.306) |
| 7 | Doctors, dentists | 0.346*** | 0.349*** | 0.345*** |
|  |  | (5.710) | (5.783) | (5.842) |
| 8 | Public health nurses | 0.379*** | 0.370*** | 0.366*** |
|  |  | (6.474) | (6.275) | (6.358) |
| 9 | Medical Technology Professionals | 0.325*** | 0.325*** | 0.320*** |
|  |  | (6.221) | (6.201) | (6.302) |
| 10 | Social welfare workers | 0.0841*** | 0.0773*** | 0.0835*** |
|  |  | (2.880) | (2.767) | (2.971) |
| 11 | Legal Professionals | -0.0254** | -0.0259** | -0.0203 |
|  |  | (-2.102) | (-2.063) | (-1.491) |
| 12 | Finance and insurance | 0.00553 | 0.00571 | 0.00768 |
|  |  | (0.293) | (0.299) | (0.397) |
| 13 | Management Business consultants | 0.0153 | 0.00957 | 0.0187 |
|  |  | (0.547) | (0.376) | (0.712) |
| 14 | Teachers | 0.00121 | -0.000529 | -0.00211 |
|  |  | (0.126) | (-0.0564) | (-0.229) |
| 15 | Religions | -0.00400 | -0.0238 | -0.0133 |
|  |  | (-0.0551) | (-0.462) | (-0.215) |
| 16 | Authors, journalists, editors | -0.00810 | -0.00443 | -0.00772 |
|  |  | (-0.380) | (-0.196) | (-0.356) |
| 17 | Artists, designers, photographers | -0.0152 | -0.0146 | -0.0161 |
|  |  | (-1.334) | (-1.270) | (-1.436) |
| 18 | Other specialist professionals | 0.0161 | 0.0139 | 0.0134 |
|  |  | (1.028) | (0.908) | (0.899) |
| 19 | General clerical | -0.00544 | -0.00710 | -0.00932* |
|  |  | (-0.942) | (-1.235) | (-1.656) |
| 20 | Accountancy | -0.00507 | -0.00527 | -0.00693 |
|  |  | (-0.567) | (-0.588) | (-0.789) |
| 21 | Production-related clerical | -0.0136 | -0.0145 | -0.0142 |
|  |  | (-1.289) | (-1.336) | (-1.323) |
| 22 | Sales clerks | -0.0123* | -0.0138** | -0.0147** |
|  |  | (-1.899) | (-2.149) | (-2.336) |
| 23 | Outdoor service | -0.00500 | -0.0123 | -0.0121 |
|  |  | (-0.166) | (-0.467) | (-0.527) |
| 24 | Transport and post clerical | -0.0268*** | -0.0288*** | -0.0308*** |
|  |  | (-2.742) | (-3.008) | (-3.298) |
| 25 | Office appliance operators | -0.0347** | -0.0400*** | -0.0426*** |
|  |  | (-2.325) | (-3.100) | (-3.579) |
| 26 | Sales workers | -0.0143** | -0.0153** | -0.0170*** |
|  |  | (-2.286) | (-2.462) | (-2.782) |
| 27 | Family Life Support and Care Service | 0.129*** | 0.129*** | 0.128*** |
|  |  | (3.862) | (3.822) | (3.760) |
| 28 | Occupational health and hygiene | 0.0690 | 0.0675 | 0.0648 |
|  |  | (1.640) | (1.617) | (1.590) |
| 29 | Food and drink cooking | -0.00269 | -0.00181 | -0.00259 |
|  |  | (-0.290) | (-0.196) | (-0.285) |
| 30 | Residential facilities and buildings | 0.0110 | 0.00744 | 0.00870 |
|  |  | (0.643) | (0.425) | (0.506) |
| 31 | Other service workers | -0.00817 | -0.00963 | -0.00948 |
|  |  | (-1.172) | (-1.393) | (-1.377) |
| 32 | Security workers | 0.0127 | 0.00840 | 0.00910 |
|  |  | (0.598) | (0.410) | (0.446) |
| 33 | Agriculture, forestry and fishery | 0.00521 | 0.00145 | 0.0112 |
|  |  | (0.306) | (0.0913) | (0.653) |
| 34 | Manufacturing process | -0.0129* | -0.0125* | -0.0139* |
|  |  | (-1.772) | (-1.695) | (-1.922) |
| 35 | Transport and machine operation | -0.00190 | -0.00279 | -0.00410 |
|  |  | (-0.149) | (-0.227) | (-0.338) |
| 36 | Construction and mining | -0.0298*** | -0.0303*** | -0.0310*** |
|  |  | (-2.595) | (-2.645) | (-2.655) |
| 37 | Carrying, cleaning, packaging | -0.0119 | -0.0138 | -0.0141 |
|  |  | (-1.325) | (-1.577) | (-1.622) |
| 38 | Other | -0.0147** | -0.0170*** | -0.0147** |
|  |  | (-2.282) | (-2.678) | (-2.305) |

**Figure A1: Average Marginal Effects for Social Capital in Table 2, Column 4 (with 95% confidence intervals)**

| Trust in other people |  |
| --- | --- |
| Reciprocity |  |
| Regional Cooperation |  |

**Figure A2: Average Marginal Effects in Table 3 (with 95% confidence intervals)**

| Trust | Reciprocity | Regional Cooperation |
| --- | --- | --- |
| Self-protection | | |
|  |  |  |
| Protect others | | |
|  |  |  |
| Safety concern | | |
|  |  |  |
| Side effects concern | | |
|  |  |  |
| No COVID-19 | | |
|  |  |  |
| Mask | | |
|  |  |  |
| No need | | |
|  |  |  |

**Figure A3: Average Marginal Effects for Social Capital in Table 4 (with 95% confidence intervals)**

| Trust in other people |  |
| --- | --- |
| Reciprocity |  |
| Regional Cooperation |  |
